# Progress and obstacles in transplantation of brown adipose tissue or engineered cells with thermogenic potential for metabolic benefits

**DOI:** 10.3389/fendo.2023.1191278

**Published:** 2023-05-16

**Authors:** Tao Zhu, Xu Chen, Shenglu Jiang

**Affiliations:** Department of Basic Medical Sciences, Taizhou Central Hospital (Taizhou University Hospital), Taizhou, China

**Keywords:** brown adipose tissue, uncoupling thermogenesis, transplantation, engineering, energy expenditure, metabolism

## Abstract

Transplantation of brown adipose tissue (BAT), engineered thermogenic progenitor cells, and adipocytes have received much attention for the improvement of obesity and metabolic disorders. However, even though the thermogenic and metabolic potential exists early after transplantation, the whitening of the brown fat graft occurs with metabolic function significantly impaired. In this review, specific experiment designs, graft outcomes, and metabolic benefits for the transplantation of BAT or engineered cells will be discussed. The current advancements will offer guidance to further investigation, and the obstacles appearing in previous studies will require innovation of BAT transplantation methods.

## Background

The obesity pandemic along with the high morbidity rate of concomitant diseases constitutes a serious danger to worldwide population health (1). Quite a number of cardiovascular, neoplastic, infectious, and autoimmune diseases have been reported to be involved in the pathological course of obesity, which significantly causes functional damage to multiple systems and a decline in the patient’s quality of life and longevity (2–4). Limited to the individual’s poor subjective initiative, keeping a balanced diet and regularly exercising to lose weight seem difficult for most people. Invasive methods such as liposuction and sleeve gastrectomy have their intrinsic risks, which definitely set restrictions on their development on a larger scale (5, 6). In addition, weight-loss drugs currently approved by the FDA, most of which are based on appetite suppression or reduction of energy absorption, such as Orlistat, usually elicit adverse reactions (7, 8). On the other hand, especially for bedridden patients with severe obesity, increasing energy expenditure in the resting state can theoretically improve metabolic status. Therefore, how to safely and effectively increase the basal metabolic rate has become the focus in the field of obesity research in recent decades (9).

There are three major types of adipose tissue in humans: white adipose tissue (WAT), brown adipose tissue (BAT), and beige adipose tissue (beige AT), as verified by anatomy, imageological examination, and histological and functional features (10, 11). WAT was firstly recognized by researchers with adipocytes in both subcutaneous and visceral depots possessing a large unilocular lipid droplet and few mitochondria and subserving the function of energy storage in the form of triglycerides (12). By contrast, BAT, primarily identified in newborns and the supraclavicular and paravertebral regions of adults, usually overexpresses uncoupling protein 1 (UCP1) in the mitochondrial membrane and is characterized by shorter cell diameter, multilocular lipid droplets, and abundant mitochondria in adipocytes (13, 14). Histologically, BAT possesses more substantial vascularization and innervation in morphology and substantially more active substrate uptake for lipid and glucose metabolism, and more lipolysis and uncoupling thermogenesis in metabolic aspects than WAT (15). Beige AT, which resembles classic BAT, can be induced within WAT depots under stimuli such as cold exposure, *in-vivo* application of β3 adrenoreceptor agonists, peroxisome proliferator-activated receptor (Pparγ) agonists, etc. (16–18). Non-shivering heat production is the most remarkable feature of BAT, which relies on UCP1 to uncouple the respiratory chain from oxidative phosphorylation and acts as an important way to increase the energy consumption without relying on muscle unit exercise in the resting state (19). In addition, BAT could also be regarded as a secretory organ to exert beneficial effects on metabolic disorders (20). It has been demonstrated that several BAT-derived molecules, among which fibroblast growth factor 21(FGF21), interleukin 6(IL6), and neuregulin 4(NRG4) are identified first, act in a paracrine or endocrine manner to regulate the metabolism of other tissues and organs (21).

Thermogenic adipose tissues, including BAT and beige AT, directly dissipate chemical energy as heat with significant weight loss in mammals and markedly improve glucose and lipid homeostasis especially after endogenous or exogenous stimulation (22, 23). As observed in many animal models with metabolic diseases, the abnormally elevated serum glucose, lipid, and other featured metabolic substrates showed nonnegligible improvement in the presence of sufficient thermogenic adipose tissues. The regained metabolic homeostasis appeared to be both long-lasting and comprehensive after further *in vivo* examination showed enhanced insulin sensitivity, glucose and lipid tolerance, and substrate uptake and consumption rate, as long as the thermogenic adipose tissues continued to function (24). In humans, positron emission tomography-computed tomography (PET-CT) scans provided researchers with explicit descriptions of BAT distribution, and statistical analysis of study cohorts indicated that individuals with detectable thermogenic BAT had lower body mass index (BMI) and lower prevalence of type 2 diabetes, dyslipidemia, coronary artery disease, cerebrovascular disease, congestive heart failure, hypertension, and other diseases associated with metabolic disorders (25, 26).

Regretfully, there is a significant decrease in the volume and activity of BAT especially in elderly or obese mammals, which is called the whitening of BAT (27). It has been validated that the reduced function of BAT can lead to obesity and other related complications (28). Multiple approaches targeted at restoring BAT function have been validated in relevant research, among which BAT transplantation seems to be an appropriate method (29–31). However, according to our previous studies, there still exist many technical difficulties in BAT transplantation, and unsatisfactory results, such as low retention rate or rapid whitening of transferred BAT, allograft induced systemic immune reaction, or disruption to the sympathetic system, might occur (32, 33). In this review, we expound recent advancements in BAT transfer, highlighting the promising application fields, alternative transplantation methods, regional or systemic risks, and possible approaches to enhance the transplantation results.

## Diseases correlated with the functional decline of BAT

BAT can function as a “heat production factory”, with the UCP1 protein in mitochondria inner membrane mediating uncoupling thermogenesis when fueling lipids and glucose (34). However, the thermogenic potential of BAT can be significantly impaired with aging or the development of obesity, which is called the whitening of BAT (35). In elderly obese individuals, BAT in specific anatomical regions showed reduced content with less vascularization and lightened brown color. In histology, the main feature of whitening BAT is the conversion of brown adipocytes to white-like unilocular cells, and the factors triggering the whitening process always lead to massive macrophage infiltration, programmed apoptosis of brown adipocytes, and a scattered crown-like structure, which can usually be seen in traditionally transferred WAT and indicate tissue necrosis (36). Regarding cytoarchitecture, the whitened brown adipocytes, surrounded by an increased number of collagen fibrils, are characterized by enlarged endoplasmic reticulum, cholesterol crystals, and some degenerating mitochondria (37). In molecular biology, the gene expression pattern of whitening BAT might show upregulated inflammasome activation, ER stress markers, and a deficiency in markers of vascularization,β-adrenergic signaling, electron transport chain, rate-limiting enzymes regulating substrate breakdown, specific membrane receptors, etc. (38–40).

The disease spectrum associated with BAT whitening is dominated by obesity and obesity-related metabolic diseases, and the therapies aiming to reverse the whitening process have been confirmed to have varying effects on these diseases by many studies (41–43). First of all, obesity is the phenotype consequence of energy intake exceeding energy expenditure in a certain time, therefore the functional decline of BAT is destined to increase the risk of obesity. Previous studies revealed that brown adipocytes can efficiently ingest substrates such as lipids, carbohydrates, and even succinic acids in blood circulation (44, 45). The classic intracellular lipid metabolism starts from the gradual lipolysis by lipolytic enzymes, such as adipose triglyceride lipase (ATGL), hormone-sensitive lipase (HSL) and monoglyceride lipase (MGL), and then the fatty acids produced can be transported into the mitochondria by special transport proteins on the mitochondrial membrane (46–48). Ultimately, the UCP1 protein on the mitochondrial inner membrane eliminates the potential energy gap inside and outside the mitochondria, resulting in the inability of high-energy protons to be transferred to energy carriers such as adenosine triphosphate (ATP) and directly converted into thermal energy instead. The thermogenic potential of BAT, through dissipating stored chemical energy as the more disordered form, is dramatic when activated (49). For an adult, the amount of BAT is generally less than 200 grams, which is an order of magnitude less than WAT. To our surprise, BAT can increase the daily energy consumption of 25 to 211 kcal after being activated by cold exposure, and the heat production of BAT is significantly enhanced by approximately 200 kcal per day after the administration ofβ-adrenergic receptor agonists. As proved by the detected volume in PET-CT scans, the importance of BAT in adults was validated by the amount of BAT being inversely correlated with body-mass index, especially in older people (50).

Even though the ability of BAT to protect against chronic metabolic diseases has traditionally been attributed to its capacity to utilize glucose and lipids for thermogenesis, it also plays a secretory role, which enables the establishment of extra protection against metabolic diseases, such as type 2 diabetes mellitus and dyslipidemia (51, 52). Most of the BAT-derived molecules, usually referred to as batokines, promote hypertrophy and hyperplasia of BAT, vascularization, innervation, and other processes that are all associated with BAT recruitment when thermogenic activity is enhanced (53, 54). The paracrine or autocrine batokines construct positive feedback to stimulate the potential of BAT. On the other hand, the batokines secreted into systemic circulation have substantial impact on targeted organs or tissues, which directly influences the progression of many metabolic diseases (55). Bone morphogenetic protein 7(BMP7), as a classical batokine, could lead to an acute decrease in food intake partly through a central rapamycin-sensitive mTOR-p70S6 kinase pathway after Intracerebroventricular administration. In addition, among a variety of batokines, FGF21, IL6 and ANGPTL8 might directly target the pancreas and improve insulin secretion and β-cell function, while NRG4 and IGF1 had been demonstrated to attenuate lipogenesis in the liver (56–58). In addition to contributing to the obese phenotype and dysfunction of glucose and lipid metabolism, whitened BAT could cause the onset of polycystic ovary syndrome, gut flora disorder, and cardiovascular diseases as well (59, 60). Therefore, BAT transfer, as an effective way to combat the whitening process, has been adopted by many researchers in the treatment of obesity and other diseases correlated with metabolic disorder.

## Transplantation of brown adipose tissue

Autologous transplantation of traditional subcutaneous WAT or its mechanical processed products was usually utilized for the reconstruction of soft-tissue defects in clinical practice (61, 62). Interestingly, the spontaneous browning of transferred WAT was observed in some studies, which did not last long and might be part of the survival mechanism where the browned subcutaneous WAT participates in adaptive tissue remodeling following grafting and contributes to adipose tissue repair under the extreme ischemic and hypoxic environment after transplantation (63–65). Of course, the temporarily transformed brown-like adipocytes did not exert much influence on overall energy expenditure and metabolism, which also highlighted the importance of browning characteristic maintenance in fat transplant with the aim of long-term metabolic improvement.

Contrary to the large reserves and easy access of subcutaneous WAT, BAT depots are of low reserves and confined to specific anatomical regions in mammals (66). Under such circumstances, the brown fat transplants mainly included *in vivo* surgically harvested BAT and engineered brown adipocytes, beige adipocytes, or preadipocytes (67, 68). Generally, the transplantation methods included autologous transplantation, syngeneic transplantation, and cross-species transplantation, with the immune response greatly eliminated by selecting the immunocompromised mammals, such as nude mice, as recipient subjects (69).

BAT obtained in mammals yielded different outcomes after transplantation because of the variation of the strain, age, donor sites, receiving sites, total transferred volume, perioperative treatment, etc. (**Table 1**) (70). According to the survival and regeneration theory proposed by Yoshimura et al., large-volume fat grafting would lead to necrosis of the core area due to the ischemic and hypoxic microenvironment in the transplanted areas, and only the transplanted fat spheres with a diameter of no more than 3.2 mm could be completely vascularized under ideal conditions (71, 72). In addition, although BAT itself has brown adipocytes with a larger surface-to-volume ratio and presents higher microvessel density than both WAT and beige AT, it exhibits the lowest retention rate after transplantation, probably due to the necrosis of the high oxygen consumption brown adipocytes and the local persistent inflammatory state (32). Therefore, the transplantation of the whole brown fat pads or large volume of mechanically processed brown fat fragments would result in massive necrosis in the core zone of the fat graft, which suggests the BAT acquired in mammals should be broken into pieces in advance, and the total transferred volume in one recipient site should be limited for better survival. In addition, the implantation of BAT fragments with extremely small volume would lead to the complete disappearance of the fat graft, where it has been previously reported that subcutaneously transferred brown fat weighing 1-3 mg did not form fat pads (73). The explanation for this outcome could lie in the insufficiency of regeneration signals, leading to failure of the recruitment and directed differentiation of stem cells, and causing clearance mediated by rapidly recruited M1 type macrophages.

**Table 1:** Previous studies of transplanted BAT or engineered cells with thermogenic potential are summarized.

Graft sources	Recipient sites	Treatments	Graft outcomes and metabolic effects
Brown fat from WT mice	Perirenal in WT mice	None	No innervation after 2 weeks. Increased cell size and lipid content. Metabolic effect not detected.
Brown fat from WT or ob/ob mice	Perirenal In WT mice or ob/ob mice	(1)Exposure to 4°C for consecutive 5 weeks (2)Exposure to 23°C or 33°C for consecutive 5 weeks (3)Exposure to 4°C for consecutive 5 weeks followed by another 3 week exposure to 23°C	(1) Long time exposure to cold temperature completely transformed lipid size and mitochondrial structure to that of host. Satisfactory innervation and vascularization. This phenomenon was not observed in host ob/ob mice. (2) Warm or hot temperature partially transformed lipid size and mitochondrial structure to that of host. Very few innervations or vascularization. (3) Cold adaptation followed by warm temperature exposure still maintained complete transformation to that of host. Satisfactory innervation and vascularization . This phenomenon was not observed in host ob/ob mice.
Brown fat from WT mice weighing 1–3 mg	Subcutaneous in WT mice	None	Subcutaneous fat pad not formed.
Brown fat fragments from rats	Intramuscular in rats	None	Intramuscular fat pad formed. Metabolic effect not detected.
Brown fat fragments from WT mice	Dorsal subcutaneous region in WT mice	None	Subcutaneous fat pad formed. A significant reduction of body weight. Increased oxygen consumption and decreased total body fat mass. Improvement of insulin resistance and liver steatosis. Up-regulation of thyroid hormone sensitivity . Increased β3-adrenergic signaling and fatty acid oxidation in WAT.
Brown fat fragments from WT mice	(1) Dorsal subcutaneous region in ob/ob mice (2) Deep into the quadriceps femoris muscle in ob/ob mice	None	Subcutaneous fat pad formed with the retention rate being 24.2% ± 3.5% and histological and gene expression pattern features showing fat transplant whitening after 16 weeks. Short-term but no long-term effect on the reduction of body mass gain, increase of energy expenditure or reduction of subcutaneous adipose tissue inflammation. Intramuscular fat pad formed with the retention rate being 22.0% ± 3.6% and histological and gene expression pattern features showing maintained BAT features after 16 weeks. Both short-term and long-term effect on the reduction of body mass gain, increase of energy expenditure and reduction of subcutaneous adipose tissue inflammation.
Brown fat fragments from WT mice	Flank subcutaneous region in WT mice	None	Compared to the transferred beige fat or white fat, the grafted brown fat showing the lowest retention rate and more severe inflammation and adipocyte apoptosis early after transplantation. Whitening of transferred BAT. Metabolic effect not detected.
Beige adipose tissue from ex vivo browning of subcutaneous WAT in WT mice	Re-implantation subcutaneously into the host WT mice.	None in this research, but could potentially act with pharmacological approaches.	Endogenous BAT increased. Metabolic effect not detected.
VEGF-A-overexpressing adipose tissue	diet-induced obese mice	None	Systemic metabolic benefits, associated with improved survival of adipocytes and a concomitant reduced inflammatory response.
Brown adipose tissue from rats	(1)PCOS rats (2)PCOS mice	None	(1)Recovered the ovarian function of PCOS rats. (2)Significantly prolonged the fertility of aging mice and did not cause severe rejection reaction, and significantly recovered ovarian functions. improved insulin resistance.
Isolated brown adipocytes or preadipocytes	Perirenal in rats	None	Fat pad not formed.
Brown adipose progenitor cells (BAPCs) from WT mice	Limb skeletal muscles in WT mice	VEGF administration	The ectopic formation of UCP1+ adipose tissue with long-term engraftment (>4 months). Combining VEGF with the BAPC transplant further improved BAT formation in muscle.
C3H10T1/2 cells	Subcutaneous in athymic mice	BMP7 administration	UCP1+ brown fat pad formed. Increased energy expenditure. Dreased body weight gain decreased.
MEFs	Subcutaneous in athymic mice	Transduced with PRDM16 and C/EBP-β	Formed brown fat pad with UCP1-positive multilocular and unilocular fat cells. Glucose uptake into fat pad. Increased basal respiration.
CRISPR-engineered human brown-like adipocytes	In thoracic-sternum region of diet-induced obese nude mice	Fed with 45% HFD for a further 4 weeks	Reconstitute functional adipocytes and activate endogenous murine BAT in mice. Facilitate glucose metabolism and thermogenesis and display long-term metabolic benefits in mice.
White adipose tissue-derived multipotent stem cells (ADMSCs) within optimized hyaluronic acid-based hydrogels	Subcutaneous implantation in WT mice	None	Distinct UCP1-expressing implants that successfully attracted host vasculature and persisted for several weeks. Elevated core body temperature during cold challenges, enhanced respiration rates, improved glucose homeostasis, and reduced weight gain.

The graft sources, recipient sites, perioperative treatments and transplantation outcomes are listed in detail.

BAT acquired from WT mice presented beneficial effects to the metabolism of recipient WT or obese mice, including the reduction of body weight, decrease of total body fat mass, and increase of energy expenditure, and improvement of insulin resistance, thyroid hormone sensitivity, and liver steatosis (74). Regretfully, the transferred BAT did not keep its thermogenic and metabolic characteristics for long, with the whitening process aggravated over time in brown fat graft especially when transferred to obese mice (75). Syngeneic intramuscular transplantation of brown fat fragments in rats showed that fat pads formed well in the recipient site based on the magnetic resonance imaging and ultrastructural studies (76). Our previous study showed that the transplanted BAT from WT mice deep into the quadriceps femoris muscle in ob/ob mice could maintain the BAT features up to 16 weeks, exerting a lasting influence on the reduction of body mass gain, increase of energy expenditure, and reduction of subcutaneous adipose tissue inflammation (33). The better microvascular foundation and more abundant secretion of paracrine factors acting as browning agents, such as irisin and other myokines in muscle microenvironment, were the possible mechanism for the long-term browning feature maintenance of intramuscular BAT transplantation (77–79). In addition, exposure to cold after transplantation enabled the brown fat graft to maintain satisfactory innervation and vascularization among adipocytes similar to that of BAT in WT mice for a longer time (80).

However, BAT resected from both diet-induced and genetically abnormal obese mice showed decreased UCP1 expression, sparse mitochondria distribution, and increased cell size and lipid content of adipocytes (81). While transplanting the whitening BAT from obese mice to WT mice, the transplant itself regained some browning features similar to that of the host but had hardly any beneficial effect on the metabolism of the host (80). Transplantation of BAT between obese or WT mice also indicated that the host environment, rather than the donor of the fat graft, determined the morphological and functional change of the transplanted BAT in the early phase after transplantation.

Autologous or syngeneic transplantation of beige adipose tissue, either induced *in vivo* by cold exposure and browning agents or from ex vivo browning of subcutaneous WAT from WT mice, showed increased endogenous BAT content and improved metabolic status early after transplantation (82, 83). Even though the retention rate of transferred beige adipose tissue was the highest compared to WAT and BAT transplantation, its thermogenic and metabolic potential was much weaker than BAT, which made it unsuitable as a fat transplant for improving metabolic abnormalities (32). Transplantation of VEGF-A-overexpressing adipose tissue from a doxycycline-inducible adipocyte-specific mouse model to diet-induced obese mice showed systemic metabolic benefits associated with improved survival of adipocytes and a concomitant reduced inflammatory response (84). This study showed the importance of rapid vascularization in transplants in the maintenance of BAT function after transplantation.

Rat-to-mouse BAT xenotransplantation, as reported recently, did not cause obvious immunorejection and significantly recovered the fertility of mice with polycystic ovarian syndrome (PCOS), accompanied by the recovery of oocyte quality, up-regulation of multiple essential genes and kinases connected to ovarian function, and the improvement of insulin resistance (85). This study provided feasibility of BAT xenotransplantation by the quantitative description of immune response, which has always been assumed to be strong by default on most occasions. In the future, research will continue to search for a BAT xenograft that elicits little immune response or drugs with the potential to dramatically reduce immune rejection of a BAT xenograft when transferred to humans.

## Transplantation of engineered thermogenic progenitor cells or adipocytes

The methods of obtaining BAT *in vivo* are limited by the inadequate storage and the inevitable large trauma to the donor sites; thus, utilizing ex vivo culturing, expansion, and engineering of thermogenic progenitor cells or adipocytes for the brown fat transplants seems like a feasible alternative choice (**Table 1**). First of all, implantation of isolated brown adipocytes or preadipocytes under the kidney in rats yielded no newly formed fat pads, which indicated that the perirenal area, lacking sufficient vascularization and cytokine secretion, might be unsuitable as the recipient site for preadipocyte or brown adipocyte transplantation (76).

Transplantation of isolated and expanded brown adipose progenitor cells (BAPCs) into limb skeletal muscles showed the ectopic formation of UCP1 positive adipose tissue with long-term engraftment and augmented energy expenditure, and combining VEGF with the BAPC transplant further improved BAT formation in muscle (86). Furthermore, a previous study had CRISPR-engineered human brown-like adipocytes transferred to the thoracic-sternum region of diet-induced obese nude mice, with the result showing facilitated glucose metabolism and thermogenesis and presenting long-term metabolic benefits in the group fed with a 45% high-fat diet(HFD) for a further 4 weeks (69). Taken together, transplantation of the engineered thermogenic progenitor cells or adipocytes favored well-vascularized recipient sites since the transplant survived the extreme microenvironment partly by the infusion of nutrients and oxygen through newly formed microvessels.

However, some engineered cell lines still showed satisfactory browning features when transferred subcutaneously into recipients. *In-vitro* cultured C3H10T1/2 mesenchymal progenitor cells were reported to be capable of changing into brown adipocytes and increasing energy expenditure and mitochondrial biogenesis, and decreasing weight gain after BMP7 administration when transplanted subcutaneously into athymic mice (87). Likewise, MEFs transduced with retroviral PRDM16 and C/EBP-β showed brown adipocyte differentiation within the formed fat pads with high expression of UCP1, active glucose uptake, and increased basal respiration after subcutaneous transplantation into athymic mice (88).

Transplantation of white adipose tissue-derived multipotent stem cells (ADMSCs) conjugated with optimized hyaluronic acid-based hydrogels into the subcutaneous region of WT mice successfully attracted host vasculature and persisted for several weeks, and functionally elevated core body temperature during cold challenges, enhanced respiration rates, improved glucose homeostasis, and reduced weight gain (89). In this study, the hyaluronic acid-based hydrogels worked as cell scaffolds for supporting the directed differentiation of ADMSCs and the eventual establishment of functional brown fat-like depots. This attempt proved the importance of the extracellular matrix (ECM) in the regeneration process after engineered adipocyte or preadipocyte transfer. Biomaterials providing extracellular scaffolds and inducing directed differentiation towards UCP1-expressing adipocytes, such as the acellular adipose matrix (AAM), might be useful for assisting the transplantation of engineered adipocytes or preadipocytes (90, 91).

## Possible methods for preventing the whitening of transferred BAT or engineered cells

First of all, the intrinsic histological and functional characteristics of the graft itself and the selection of the transplant recipient sites all affect the biological performance of the graft, including the alteration of UCP1-mediated thermogenesis and systemic metabolic activity (92). BAT obtained from aged or obese individuals exhibited impaired thermogenic and metabolic potential and produced much fewer metabolic benefits to the host after transplantation; thus, selecting young and healthy donors could improve transplantation results (93). In addition, the engineering method of BAT or even isolated adipocytes and preadipocytes aiming to enhance the thermogenic potential, including the application of CRISPR and iPSC technology, showed varying graft destiny and manifestations of its browning characteristics after transplantation. Diverse engineering methods have endowed BAT or brown fat-like cells with greater thermogenic potential, which has drawn much attention from researchers recently (94).

**Figure 1:**
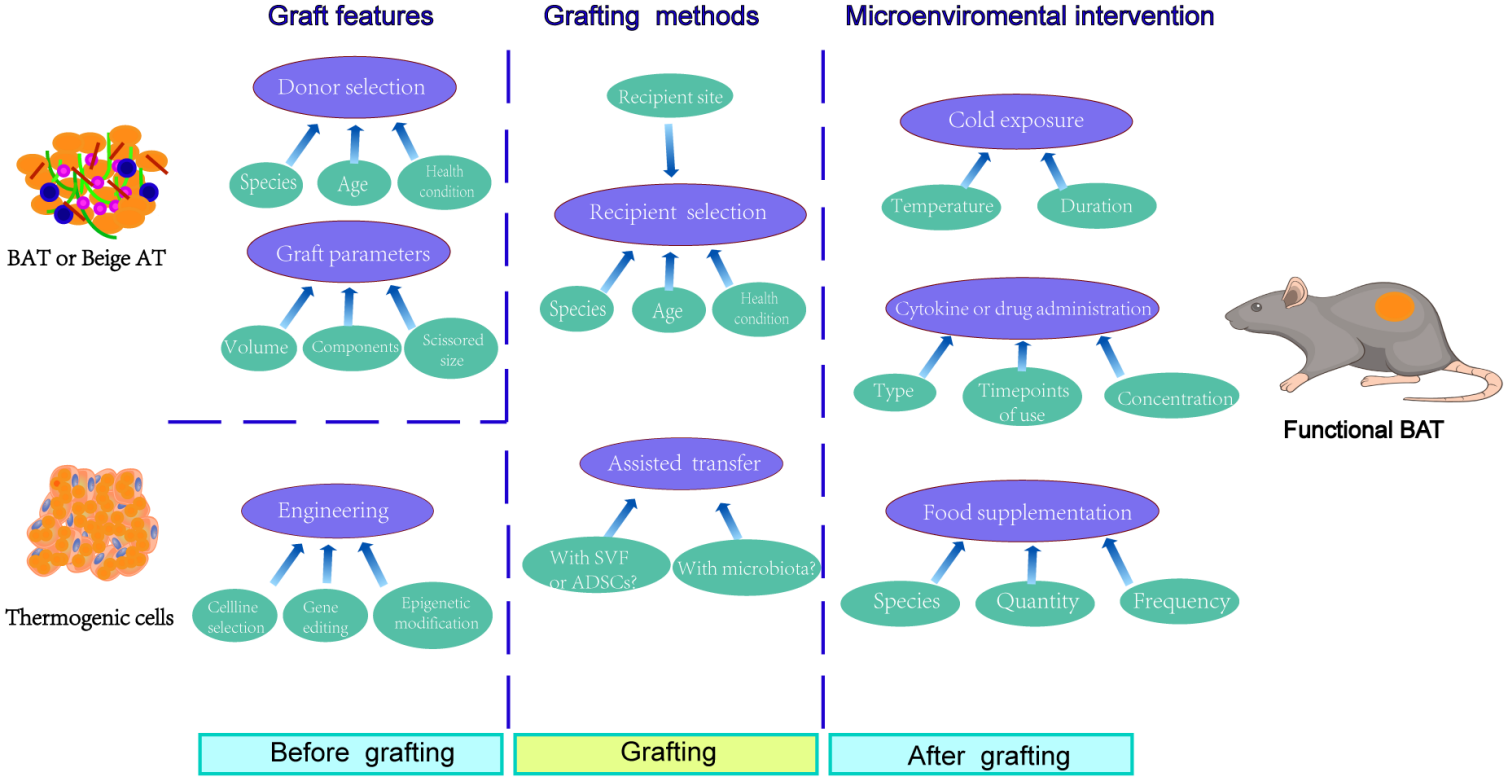
Factors involved in graft outcomes and function maintenance of transferred BAT or thermogenic cells. Before grafting, the graft itself dramatically determined the transplantation outcomes, which mainly involved the donor selection and the mechanical pretreatment of adipose tissue. As for thermogenic cells, the engineering methods, including cell line selection, gene editing, and epigenetic modification, affected the transfer results. During grafting, the recipient selection was also relevant to the graft outcomes, especially for the choice of recipient site. In addition, the simultaneous transplantation of stromal vascular fraction (SVF), adipose derived stem cells (ADSCs), or gut microbiota from healthy donors seemed like adoptable approaches for improving grafting results. After grafting, however, the microenvironmental intervention using cold exposure, cytokine or drug administration, or even specific food supplementation provided a promising way for the function maintenance of transferred BAT or thermogenic cells.

In addition, the well-vascularized and active cytokine secreting recipient areas facilitated the survival and maintenance of browning features of brown fat graft (95). Therefore, compared to perirenal or subcutaneous transplantation, intramuscular transplantation of either BAT resected *in vivo* or adipocytes and preadipocytes engineered *in vitro* showed better retention and prolonged BAT feature maintenance (**Figure 1**).

Long-term cold exposure was adopted by many researchers for the induction of beige adipose tissue or the enhancement of browning features of BAT (96). In several studies conducted on mammals, recipients exposed to cold after BAT transplantation manifested the prolonged browning feature maintenance of fat graft and systemic metabolic benefits (80, 97). In addition, local injection or oral administration of browning agents, such asβ3 adrenergic receptor agonists, PPARγ agonists, estrogen analogs, etc., could promote the re-browning of transferred brown fat undergoing a whitening process (98, 99). In addition, local administration of growth factors and small molecule nutrients to recipient sites might promote the vascularization and regeneration of newly developed brown adipose tissue (100, 101). Sufficient uptake of food supplemented with nutrients with browning potential, such as capsaicin, synephrine alkaloids, etc., might help the maintenance of browning features of transplanted brown fat (102, 103). However, these approaches affect not only the transferred brown fat but also the function of BAT and WAT in the recipient and cause a multi-systemic response. Consequently, the overall functional change of target organs should be monitored in the practice of brown fat grafts for better risk control.

Combined transplantation of BAT and adipose-derived stem cells (ADSCs) might help the survival and regeneration of fat grafts since the transferred ADSCs have exhibited the potential to differentiate into vascular cell components or directly into mature adipocytes and to secrete paracrine cytokines or chemokines for the further recruitment and differentiation of stem cells from the host in previous research regarding the technology of cell-assisted lipotransfer (CAL) (104–106). Another recent discovery in the field of BAT research was that the gut microbiota modulated the metabolism and activity of BAT, and that depletion or imbalance of gut microbiota impaired thermogenesis of BAT (107–109). Under such a remarkable regulatory relationship, it would be reasonable to conduct gut microbiota transfer simultaneously with BAT transplantation for pronged thermogenic and metabolic benefit maintenance.

## Conclusion

Unlike the utilization of subcutaneous WAT in volume augmentation for esthetic and reconstructive surgery, the preserved thermogenic and metabolic potential of transferred BAT or engineered adipocytes and preadipocytes inspired researchers to tentatively apply transplantation of thermogenic adipose tissue or engineered cells for metabolic improvement. Brown adipose tissue *in situ* functions as a heat production factory with the UCP1 protein mediating uncoupling thermogenesis based on the adequate vascularization for sufficient substrate and oxygen supply and appropriate stimulation of certain cytokines, such as BMP7 and PRDM16, for directed differentiation and functional protein expression of progenitor cells. Unfortunately, the brown fat graft was in an extreme microenvironment where the oxygen and nutrient supply was obviously insufficient and the signaling molecules were differently expressed.

Whitening of the transferred BAT or engineered adipocytes and preadipocytes remain a barrier to the long-term improvement of energy expenditure and metabolism. The specific mechanisms and possible solutions for whitening of transferred BAT are still poorly understood. Therefore, it is necessary to analyze recent progress in the improvement of brown fat graft methods and to further elucidate the possible influencing factors in order to prevent the whitening of transferred BAT or engineered brown fat-like cells and create a mobile metabolic factory for sustainable thermogenesis.

## Author contributions

TZ and SJ conceived and wrote the manuscript, while XC searched the database and provided some relevant literature. All authors contributed to the article and approved the submitted version.
